# Telerehabilitation Applied By Physiotherapists To Older Adults In Brazil - A Pilot Survey

**DOI:** 10.1093/geroni/igaf122.2450

**Published:** 2025-12-31

**Authors:** Roberta Oliveira Bueno de Souza, Fabiana Cassales Tosi, Judith Deutsch, Ruth Melo, Jose Eduardo Pompeu

**Affiliations:** University of Sao Paulo, Sao Paulo, Sao Paulo, Brazil; University of Sao Paulo, Sao Paulo, Sao Paulo, Brazil; Rutgers University, New Brunswick, New Jersey, United States; Universidade de São Paulo, São Paulo, Sao Paulo, Brazil; University of Sao Paulo, Sao Paulo, Sao Paulo, Brazil

## Abstract

This study aimed to characterize the profile and adherence rate of Brazilian physiotherapists who utilize telerehabilitation (TF) in the care of community-dwelling older adults and evaluate their satisfaction with the intervention. A cross-sectional observational study was conducted through an online survey between August and December 2024, using convenience sampling. The questionnaire investigated the physiotherapists’ profile, characteristics of TF, and their satisfaction with the intervention. A total of 59 responses were obtained, with 55.9% from physiotherapists working with older adults. Most respondents were female (90.9%), with a mean age of 41.0±10.6 years, residing in the Southeast region (78.8%), and 78.8% had more than 5 years of experience. Additionally, 84.8% held a specialist title, and 78.8% adopted TF. Most TF sessions were conducted individually (55.2%), lasted between 46 and 60 minutes (65.5%), and utilized computers and/or smartphones (48.3%) with commercial softwares (82.8%). The synchronous modality was the most commonly used (96.6%), which was applied during treatment (82.8%). Regarding satisfaction perception, 69.0% rated it as satisfactory or very satisfactory. In conclusion, the use of TF in community-dwelling older adults in Brazil has a good adherence rate and is considered satisfactory or very satisfactory by Brazilian physiotherapists. Telerehabilitation for the Brazilian older adults is delivered individually and synchronously during treatment, using simple and accessible technologies. However, a larger sample is needed to enhance the robustness and generalizability of these results.

